# Meta-research: Why research on research matters

**DOI:** 10.1371/journal.pbio.2005468

**Published:** 2018-03-13

**Authors:** John P. A. Ioannidis

**Affiliations:** 1 Meta-Research Innovation Center at Stanford (METRICS), Stanford University, Stanford, California, United States of America; 2 Department of Medicine, Department of Health Research and Policy, and Department of Biomedical Data Science, Stanford University School of Medicine, Stanford, California, United States of America; 3 Department of Statistics, Stanford University School of Humanities and Sciences, Stanford, California, United States of America

## Abstract

Meta-research is the study of research itself: its methods, reporting, reproducibility, evaluation, and incentives. Given that science is the key driver of human progress, improving the efficiency of scientific investigation and yielding more credible and more useful research results can translate to major benefits. The research enterprise grows very fast. Both new opportunities for knowledge and innovation and new threats to validity and scientific integrity emerge. Old biases abound, and new ones continuously appear as novel disciplines emerge with different standards and challenges. Meta-research uses an interdisciplinary approach to study, promote, and defend robust science. Major disruptions are likely to happen in the way we pursue scientific investigation, and it is important to ensure that these disruptions are evidence based.

Science, like all human endeavors, is prone to biases. Yet science can assess its own methods, reporting, reproducibility, evaluation, and incentives [1]. A relatively new discipline, called meta-research, covers a wide range of theoretical, observational, and experimental investigations designed to study research itself and its practices. The objective is to understand and improve how we perform, communicate, verify, evaluate, and reward research [1].

Before elaborating on a discipline that studies biases, I should disclose some of my own. First, all scientists are meta-researchers to some extent, though most usually work on focused subject matter disciplines. And though the advice of my early lab mentors—“focus, focus, focus”—still rings in my ears, the piles on my desk and the files in my computers can be notoriously unfocused. I don’t have attention-deficit disorder, but plain unconstrained curiosity. What attracted me to science was its vastness and diversity. In my early training years, I enjoyed roaming in libraries in Athens and Boston, discovering scientific journals with fancy names, encountering intriguing articles, drifting from my initial search. Without yet realizing it, I was interested primarily in research itself apparently, much as others were interested primarily in *Caenorhabditis elegans*, volcanic eruptions, or automata.

Science and its literature is a marvelous maze of data, arguments, biases, errors, and the greatest achievements of humans. What can be more rewarding to study scientifically? Thirty years later, I still feel like a researcher-in-training—actually, in early training—barely scratching the surface. However, much has changed. Thirty years ago, articles had to be handpicked like flowers one by one from their journal shelves and photocopied one page at a time. Now, one can text mine a million articles overnight. Good research, however, still takes time and focus. Take, for example, a recent project I worked on with my friend David Chavalarias. We text mined 12,821,790 abstracts and 843,884 full-text articles. We initially joked that it would take two days max. Eventually, it took four years of work with innumerable iterations, meticulous corrections, and repeated downloads.

My other personal bias is a heightened interest in methods rather than results. Result narratives are supposedly always exciting. I find them unbearably boring. Conversely, methods typically are missing in action, left unsung, or hidden in small print. Many researchers hope to clarify how to do experiments chatting in corridors or conferences. Study design and analysis are still mostly taught (if at all) in statistics-lite courses. Most of us have mastered how to write papers through reading other (mostly poorly reported) papers. We freely volunteer peer review but lack formal training on how to do it. In many fields, issues surrounding reproducibility were dormant until recently.

Science remains the key driver of human progress, yet we have little evidence on how to best fund science and incentivize high-quality work. We do know that leaving research practices to serendipity, biasing influences, methodological illiteracy, and statistical innumeracy is inefficient. Science needs science to avoid wasted effort and optimize resources. Amateur approaches face the current gigantic magnitudes of the research endeavor. Google Scholar currently includes about 180,000,000 documents, accruing approximately 4,000,000 new papers annually [2]. Along this universe of visible (published) matter, dark matter abounds; probably most observations and data analyses remain unpublished. Ulrich’s directory includes more than 40,000 refereed academic journals, and this is probably an underestimate [3]. Thousands of journals follow predatory practices or have uncertain value. The Science, Technology, Engineering, and Math (STEM) publishing business market size ($28 billion) roughly equals the National Institutes of Health (NIH) budget. Webometrics lists 26,368 research-producing universities [4], and many other entities generate research. Probably 100,000 biomedical conferences happen annually [5]. Global Research and Development (R&D) investment recently exceeded $2 trillion per year. Industry has the lion’s share, while public funding is limited for basic research and it is even more sparse for evidence-based evaluation research. Financial conflicts may shape research agendas, results, and interpretations [6]. Consider that the $1 trillion tobacco industry still runs “research” on its products despite killing millions of people who use them as directed. Big Pharma, another behemoth of similar financial magnitude, but which probably saves lives (albeit often at high cost), has to sponsor most research on its own products. Understanding who should do what and how in research needs better study.

Science is no longer the occupation of few intellectual dilettanti. Millions (co)author scientific papers. Even more people participate in research. Currently, health record databases engulf hundreds of millions of individuals. Social media databases generate the possibility of using data on billions—active monthly Facebook users, for example, exceeded 2 billion by July 2017.

Currently, generated research data are massive but also fragmented and often nontransparent. Full data sharing and preregistration of protocols are still uncommon in most fields [7]. We need to understand whether results and inferences are correct, modestly biased, or plain wrong. Comparing patterns of data and biases across the vast number of available studies, one can help answer this important question [8]. We have mapped 235 biases in biomedical research alone [9]. With increasing research complexity, multifarious choices emerge on how to design studies and analyze data. With 20 binary choices, 2^20^ = 1,048,576 different ways exist to analyze the same data. Therefore, almost any result is possible, unless we safeguard methods and analysis standards. Surveys show that questionable research practices are used by most scientists: not fraud (which is rare) but “cutting corners” to achieve more interesting-looking results [10]. Understanding the boundaries between bias and creative exploration is important. Efforts to reproduce high-profile studies have shown high rates of nonreproducibility [11] and most scientists agree that a reproducibility crisis exists [12]. Meta-analyses—efforts to combine all data on a given question—become increasingly popular but face their own problems and biases [13].

How should a scientist best train, work, collaborate, and contribute to scientific and broader communities? Researchers spend most of their time on grants [14] and administrative chores of unclear utility. Journal peer review takes another 64 million hours annually for biomedical papers alone [15]. Justifiably, we all despise bureaucracy and obstructions. Poor research practices make things worse.

Thousands of new scientific fields emerge, merge, split, and evolve [16]. Different disciplines may differ in research standards and challenges (Box 1). Meta-research can help us disseminate efficient research practices and abandon wasteful ones. Publication and peer review models, scientific education, funding, and academic reward systems need to adapt successfully to a rapidly changing world. Some predict [17] that even researchers may disappear within decades, replaced by artificial intelligence. While this sounds extreme, several aspects of current “business as usual” in research will face disruption. Even 1% improvement in the yield and translation of useful discoveries effected through better research practices reflects value equivalent of many Nobel or Breakthrough prizes.

**Box 1. Features of research practices, opportunities, and threats that vary across fields.**

- Type of research, designs, tools, and statistical methods
  ◦ Type of mix of research (basic, applied translational, evaluation, implementation)
  ◦ Types of study designs commonly used or misused
  ◦ Types of experimental/measurement tools commonly used or misused
  ◦ Types of statistical methods commonly used or misused

- Biases and questionable/detrimental practices
  ◦ Types of common biases encountered and whether they are easy to fix or not
  ◦ Extent of use of methods to prevent or correct for biases
  ◦ Prevalence of different types of questionable/detrimental research practices

- Targeted effects and signals
  ◦ Distribution of effect sizes observed
  ◦ Typical heterogeneity of results across studies
  ◦ Proportion of results that are true, exaggerated, or entirely false
  ◦ Reputational impact for bias or wrong, refuted results

- Publication and peer review practices
  ◦ Proportion of studies and analyses that are published
  ◦ Number and types of available publication venues
  ◦ Implementation of prepublication peer review (e.g., preprints)
  ◦ Implementation of postpublication peer review
  ◦ Extent from adoption of various research reporting standards

- Scientific workforce standards
  ◦ Commonly accepted authorship and contributorship norms
  ◦ Extent of adoption of team science and consortia
  ◦ Type of training for scientists in the field
  ◦ Extent of methodological and statistical literacy/numeracy

- Replication and transparency standards
  ◦ Extent and enforcement of preregistration of protocols
  ◦ Extent of use of replication studies
  ◦ Extent of use of exact replication versus corroboration or triangulation
  ◦ Extent of sharing of primary raw data and/or processed data
  ◦ Extent of sharing of software and code
  ◦ Extent and types of evidence synthesis used

- Reward structures and standards
  ◦ Main funders (government, industry, other) and types of studies that they fund
  ◦ Project-based versus person-based funding
  ◦ Mix and interplay of institutions performing research (university, industry, other)
  ◦ Types of metrics and criteria used for assessing researchers and institutions

- Conflicts
  ◦ Typical conflicts of interest operating in the field
  ◦ Completeness of disclosure of conflicts of interest

- Public interface
  ◦ Extent and fidelity of dissemination of research findings to the general public
  ◦ Extent of public misperceptions about the field
  ◦ Threats from antiscience advocates attacking the field

Meta-research is interdisciplinary. For example, it benefits from better tools and methods in statistics and informatics. Complex issues of behavior change converge on modeling, psychology, sociology, and behavioral economics. Newly introduced, sophisticated measurement tools and techniques in various disciplines introduce new, peculiar errors and biases; their understanding requires combining expertise in biology, bioengineering, and data sciences. Properly communicating science and its value requires combining expertise in multiple fields and has become increasingly critical nowadays, when mistrust of science runs high and multiple interests hold a stake in influencing research results. Some interests set out to manipulate science and cause damage when their intentional bias pollutes the scientific record (e.g., tobacco companies or climate change deniers). Meta-research may be our best chance to defend science, gain public support for research, and counter antiscience movements. It may help provide a correcting mechanism closer to real time than the self-correcting scientific process that otherwise may take much longer.

Moreover, bird’s-eye metaviews of science are not separate and detached from focused field-specific research. In my experience, inspiration for new projects has often come from mistakes, shortcomings, or difficulties that I encountered while doing field-specific research. It is sometimes difficult to convey a message that something is wrong. However, it is paradoxically easier when the message says that thousands or millions of papers are doing something wrong rather than arousing personal animosity for a single failed paper. It is also easier when the constructive critique comes from within a field, recognized as necessary improvement rather than intrusion. Learning by collaborating with researchers in diverse disciplines and trying to understand the daily challenges in a specific field can be a highly rewarding experience for a meta-researcher. We need scientific curiosity but also intellectual humility and commitment to improve our efforts.

## Abbreviations

NIH: National Institutes of Health
R&D: Research and Development
STEM: Science, Technology, Engineering, and Math
